# Primary Care Provider Counseling Practices about Adverse Drug Reactions and Interactions in Croatia

**DOI:** 10.3390/jcm7090231

**Published:** 2018-08-22

**Authors:** Nikola Raguz Lucic, Jelena Jakab, Martina Smolic, Ana-Maria Milas, Tea Omanovic Kolaric, Vjera Nincevic, Kristina Bojanic, Kristina Kralik, Maja Miskulin, George Y. Wu, Robert Smolic

**Affiliations:** 1 Faculty of Medicine, University of Osijek, Josipa Huttlera 4, HR-31000 Osijek, Croatia; nikola.rlucic@gmail.com (N.R.L.); jelena.jakab@mefos.hr or jelena.jakab@fdmz.hr (J.J.); martina.smolic@mefos.hr (M.S.); milas.anam@gmail.com (A.-M.M.); tomanovic@mefos.hr (T.O.K.); vnincevic@mefos.hr (V.N.); bojanic.kristina@gmail.com (K.B.); Kristina.kralik@mefos.hr (K.K.); maja.miskulin@mefos.hr (M.M.); 2 Faculty of Dental Medicine and Health, University of Osijek, Crkvena 21, HR-31000 Osijek, Croatia; 3 Division of Gastroenterology-Hepatology, Department of Medicine, University of Connecticut Health Center, 263 Farmington Avenue, Farmington, CT 06032, USA; wu@uchc.edu; 4 Division of Gastroenterology-Hepatology, Department of Medicine, Osijek University Hospital, Josipa Huttlera 4, HR-31000 Osijek, Croatia

**Keywords:** drug interactions, adverse drug reactions, primary care providers, attitudes, counseling

## Abstract

Background: Prescribing medications is one of the most common medical decisions that is made by primary care providers (PCPs). In the Republic of Croatia, PCPs hold a key position in prescribing and evaluating the medications that are provided for patients. Accordingly, providing advice for patients regarding the potential adverse drug reactions (ADRs) and drug-drug interactions (DDIs) is frequently the responsibility of the PCPs. The aim of the current study was to assess the knowledge, attitudes, and counseling practices of PCPs regarding drug interactions and adverse effects. Methods: After enrolling 195 PCPs that were selected at random, a survey was conducted while using an anonymous questionnaire that was created based on previously published studies, adjusted in a way that includes the most commonly prescribed medications in Croatia. Results: Of the 10 questions on knowledge about DDIs and ADRs, the median number of correct responses by PCPs was 5 (interquartile range 4 to 7). More than half of respondents (56%) agreed with the claim that knowledge of drug side effects facilitated their work in family medicine. Almost all of the respondents (92.8%) explained side effects and drug interactions to special groups of patients (pregnant women, elderly patients etc.). Conclusion: The results show a need for additional education in the field of drug prescribing. However, PCPs were aware of the importance of counseling practices about adverse drug reactions and interactions and counseling practices among special patients populations are satisfactory.

## 1. Introduction

Primary health care practice requires broad skills and knowledge, a holistic approach, and a good patient-doctor relationship [1]. Prescribing medications is one of the most common medical decisions made by primary care physicians (PCPs) and represents a challenge for both physicians and patients, as drugs intended to improve the health condition can also cause adverse drug reactions (ADR) or interactions with other drugs. This is particularly important with the advent of new and powerful agents that often have increased side-effects that accompany their increased potency. Similarly, new drug combinations that are used especially with comorbidities, as in the elderly, increase the likelihood of drug-drug interactions (DDIs) [2]. ADRs are mostly caused by inappropriate medication use in older patients [3]. According to the 2014 World Health Organization (WHO) data, 19% of the population of the Republic of Croatia is 65 years old or above [4]. Elderly often have coexistent chronic conditions that affect patient quality of life and result in polymedication [5]. Meta-analysis of observational studies on hospitalizations caused by ADR showed that elderly people were four times more likely to be hospitalized due to ADR-related problems than the younger patients, and most of these hospitalizations could be avoided [6]. It has been shown that most PCPs know that close attention is required when prescribing many drugs to a patient. However, it is often difficult to oversee the proper use of multiple medications due to the complexity of potential reactions and interactions [7].

Because of the organization of national health care in Croatia, PCPs hold a key position in prescribing medications for patients because current law allows only for PCPs working under Croatian Institute for Health Insurance contract to prescribe drugs that are covered by the health insurance [8,9]. The main part of primary health care, general/family medicine, is carried out by medical doctors who graduated from medical school and passed the state exam, and general/family medicine specialists who finished four years of specialization in family medicine. PCPs prescribe drugs recommended by specialists, according to their professional judgement, the availability of a drug on the market, and patient preference and previous experience with certain drugs. The aim of this study was to evaluate knowledge about ADRs and DDIs, as well as attitudes and practice regarding counseling.

## 2. Experimental Section

### 2.1. Study Tool

An anonymous self-administered 46-item questionnaire was used to explore knowledge, attitudes, and practice (KAP) among medical doctors working in the primary care in Croatia. The questionnaire contained questions that are similar to the ones included in previous local and international studies [10,11,12,13] adopted to the questions about the most prescribed drugs in Croatia and to the ones with the most harmful side effects or significant drug interactions [14,15,16]. The first version of the questionnaire had 39 questions that were divided in three major sections. The first section focused on the physicians’ demographics (gender, age, place of work, level of education, years of service); the second section consisted of 10 questions to document the knowledge about DDIs and ADRs in the PCP’s setting, and the third section comprised attitude related questions and identified practices regarding counseling about ADRs and DDIs.

### 2.2. Validity and Reliability of the Study Tool

The first draft of the questionnaire was reviewed by a panel of professionals, which consisted of three pharmacology professors with experience in drug-drug interactions and one epidemiology professor with experience in a survey-type research. Before the distribution, this modified version was pilot tested among 30 PCPs in Health Center Dakovo, Croatia to assess the content and validity of the tool. In concordance to reliability analysis, the final version of the questionnaire had 46 items. The main difference between the first and final version of the questionnaire was that attitude-related questions were divided in two separate sets, one for ADRs and the other for DDIs. Data obtained from the pilot study were excluded from the reported study results.

### 2.3. Ethical Approval

The study protocol was approved by the Ethical Committee of the Faculty of Medicine Osijek, Croatia (Ethical Approval Code: 602-04/18-08/07; file number: 2158-61-07-18-43).

### 2.4. Study Design and Sample Recruitment

A cross-sectional questionnaire study was performed on 195 PCPs from all 21 (randomly selected) counties of Croatia. The questionnaire was distributed as an online survey. PCPs were invited to participate by electronic mail. Data collection took place over three months (February to April 2017).

To visualize the middle effect in the numeric variables between independent groups of respondents, with a level of significance of 0.05 and strength of 0.8, the minimum required sample size was calculated to be 180 subjects (calculation made while using G*power software version 3.1.2; Franz Faul, University of Kiel, Kiel, Germany).

### 2.5. Data Analysis

Categorical data were represented by absolute and relative frequencies. Numerical data were described by arithmetic mean and standard deviation in case of normal distribution and by median and interquartile range in case of deviation from normal distribution. The variance of the category variables was tested by the Chi-square test and Fisher’s exact test. The differences between variables in two independent groups were tested by Student’s *t*-test and Mann-Whitney’s Test. The differences between three and more groups were tested by variance analysis (ANOVA) or Kruskal-Wallis test. The correlation between numeric variables was evaluated by the Pearson correlation coefficient and Spearman’s correlation coefficient ρ (rho). *p* < 0.05 was considered to be statistically significant. The analysis was conducted while using the MedCalc Statistical Software version 14.12.0 (MedCalc Software bvba, Ostend, Belgium; http://www.medcalc.org; 2014).

## 3. Results

### 3.1. Demographic Characteristics of General Practitioners

A total of 195 general practitioners participated in this study. The demographic details of the participants are summarized in Table 1. In further statistical analyses, we compared responses that were given by medical doctors who passed the state exam, and general/family medicine specialists.

The median age of the participants is 51 years (interquartile range 31 to 56 years), ranging from 25 to 65 years.

### 3.2. PCP Knowledge of Drug-Drug Interactions

The median of the correct responses of PCPs with regard to DDIs and ADRs was 5 (interquartile range 4 to 7) out of 10 questions (Table 2).

According to the number of correct answers, there are no significant differences in gender, place of work, or length of work experience. However, the number of correct answers, median 6 (interquartile range 4 to 7), given by general/family medicine specialists was significantly higher (Kruskal Wallis test, *p* = 0.01) comparing to medical doctors who passed state exam only (median number of correct answers = 5) (Table 3).

### 3.3. Attitudes and Practices of PCPs in Advising Patients about Drug Interactions and Adverse Effects

According to the results of our survey, 75 (38.5%) participants were able to explain side effects and drug interactions to all patients during their daily routine, while 92.8% PCPs gave information only to special groups of patients (pregnant women, elderly patients, etc.). The most significant obstacle to counseling on interactions and side effects was a lack of time, 84 (43.1%) (Table 4).

Evaluation of attitudes towards counseling about interactions and side effects was assessed through 21 statements. The statement that was least agreed with was that an elderly patient who takes more than eight types of drugs should be referred to a clinical pharmacologist. Most respondents, 109 (56%) agreed with the claim that knowledge of drug side effects facilitates their work in family medicine and 92 (47.2%) participants agreed that multiple drug use could be harmful to the patient (Table 5).

## 4. Discussion

The results of our study showed that median of correct responses was 5 (interquartile range 4 to 7) out of 10, emphasizing the need for additional forms of education of PCPs, and the importance of raising awareness about the harmful effect of inadequate prescribing.

The results of our study are similar to other previously published studies evaluating prescribers’ knowledge about DDIs in the United States (US), one of which conducted on 263 clinicians practicing within a Southern California Veterans Affairs health care system [17]. Another was conducted on 950 randomly selected US prescribers who prescribed one or more medications that could have caused a DDI [7]. In these studies, the physicians correctly recognized approximately 50% of drug-drug pairs, interacting combinations, and contraindicated pairs. In addition, there have been other non-Croatian studies that have shown that the awareness about pharmacovigilance regarding ADR, and DDIs is positively correlated to knowledge and practice of pharmacovigilance in India [18]. Another important area, especially in polypharmacy, is a good relationship between specialists in hospital and family medicine specialist. Only 26% of participants in our study agreed with the claim that specialists in hospitals think about the possible interactions and adverse effects when prescribing their medicine. Accordingly, most of them considered that preventing polymedication was their duty, an opinion similar to the one found in Belgian study on 65 GPs who feel that their role as a ‘gatekeeper’ is to control the type and quantity of medication used [19]. These reports support the conclusion that the findings in the current study are more broadly applicable, and not limited to the local population in Croatia.

By analyzing according to the characteristics of correspondents, a statistically significant difference was found regarding median values of correct answers, and the level of education, with the most accurate answers being provided by the group of family medicine specialists. Such a result can be explained by the fact that medical doctors who passed state exams often start working in primary health care right after graduating from medical school, whereas general/family medicine specialists have more experience in drug prescribing.

In order for the patient to know all the benefits or consequences of using the drug, it is essential for his PCP to give the patient all the information about the possible side effects and interactions of the prescribed drug. Giving verbal or written information can improve adherence to treatment, especially in the context of complex drug regimens [20]. As the most significant obstacle to conducting a consultation on interactions and side effects of prescribed medicines in their daily work with patients, 43.1% PCPs reported lack of time. According to another study using also anonymous online questionnaire, only 34% of patients and 40% of pharmacists working in South Korea were satisfied with the level of medical counseling [21], implying that a lack of time was a major problem for both groups.

The knowledge of drug pharmacokinetics, ADRs and DDIs may be helpful in preventing the harmful effects of drugs on the human body [22]. While assessing attitudes about ADR and DDIs our correspondents mainly stated that they have an important role in pharmacovigilance, but they are not completely satisfied with their knowledge in that field. More than half of our participants agreed with the statement that good knowledge in pharmacovigilance facilitates the work in everyday practice. As in other studies, comprised in a synthesis of qualitative studies by Bokhof, our participants were aware of the fact that improving their knowledge in ADR could prevent the morbidity and mortality of patients, and lead to decreased exposure to ADR due to polypharmacy [23]. Developing mechanisms for evaluating the safety of drugs in clinical use is vital for prevention of drug-related harm. The first step to improve prescribing practice is education. Some countries have developed strategies and national programs in order to provide more training courses and education classes for PCPs. Working environment is also important, because PCPs need more individual time with each patient, in order to provide as much important information as clearly as possible, and to have a better insight in patient history of morbidity and medications. There is also a need for more staff, which would result in improved work quality [24]. One study suggested that it could be beneficial to improve curriculum in order to prepare young doctors to handle the transition from basic knowledge about pharmacovigilance to the application of one in clinical practice [25]. Furthermore, the trend towards developing electronic health records (EHR) should be emphasized [26]. The use of EHR can improve pharmacovigilance, and complement spontaneous reporting systems. It can also provide a complete overview of the prescriptions by different health care providers [27]. Theoretically, ADRs due to DDIs and the duplication of therapy could be eliminated, given the fact that over 30% of ADRs are caused by DDIs.

To the best of our knowledge, this is the first study in Croatia with regard to PCP counseling and knowledge about ADRs and DDIs. Consequently, this study could help analyze various factors that would induce or distract the individual physician from conducting such counseling in the affected population.

The limitations of our study can be attributed to the design of the study, because it is difficult to establish causality with a cross-sectional study. Another limiting factor is that our questionnaire analyses depended on self-reported data that are often influenced by current mood and daily workload. Also, respondents are prone to giving socially acceptable responses rather than giving their actual opinions. However, findings of self-reported studies are still considered to be as valid as more extensive and expensive tests [28].

## 5. Conclusions

This study showed that majority of PCPs in Croatia understand the importance of good knowledge and counseling regarding ADRs and DDIs in primary health care setting. However, this study highlights the need for an additional education to enhance skills for drug prescribing to improve communication with patients and many other elements of the prescribing process in order to reduce morbidity and mortality related to drug interactions and adverse effects. To enhance the prescribing practice, it would be helpful to organize an additional education. Although improvement in working environment with more time allowed for counseling patients would be ideal, in most situations, that solution is not viable. However, good practice and positive attitudes of our PCP regarding the counseling about DDIs and ADRs are of great value and contribution to primary health care in Croatia.

## Figures and Tables

**Table 1:** Demographic characteristics of participants.

	Number (%) of Correspondents
Gender	
Male	38 (19.5)
Female	157 (80.5)
Level of education	
Medical doctors who passed the state exam	97 (49.7)
General/family medicine specialists	90 (46.2)
Other specialties	8 (4.1)
Location of practice	
City	95 (48.7)
Regional center (Zagreb, Split, Rijeka, Osijek)	40 (20.5)
Countryside	60 (30.8)
Inland	147 (75)
Coastal area	48 (25)
Working experience	
Up to 4 years	57 (29.2)
5–9 years	13 (6.7)
10–14 years	19 (9.7)
15 or more years	106 (54.4)

Data are presented as number and percentages of participants in the study according to demographic characteristics.

**Table 2:** Median of the correct answer.

The Number of Questions	Median of the Correct Answers	Interquartile Range
10	5	4–7

Table shows median and interquartile range of correct answers out of total number of questions regarding knowledge about drug interactions and side effects.

**Table 3:** Mean values of correct answers according to participant’s characteristics.

	Median (Interquartile Range) of Correct Answers	*p* *
Gender		
Male	5 (4–6)	0.22 *
Female	5 (4–7)	
Level of education		
Medical doctors who passed the state exam	5 (4–6)	0.01 *
General/family medicine specialists	6 (4–7)	
Location of practice		
City	5 (4–7)	0.22 ^†^
Regional center (Zagreb, Split, Rijeka, Osijek)	5 (4–6)	
Countryside	6 (4–7)	
Working experience		
Up to 4 years	5 (4–6)	0.21 ^†^
5–9 years	5 (4–6)	
10–14 years	6 (5–7)	
15 or more years	5 (4–7)	

Data are presented as medians and interquartile range of correct answers regarding knowledge about drug interactions and side effect in relation to demographic characteristics. * Mann Whitney; ^†^ Kruskal Wallis.

**Table 4:** Informing patients about side effects and drug interactions.

Question	Number (%) of Correspondents
**In my daily work with patients, I always advise patients about side effects and drug interactions**	
No	120 (61.5)
Yes	75 (38.5)
**In my daily work with patients, I only advise special groups of patients (pregnant women, children, elderly patients) about side effects and drug interactions in the following way:**	
I give oral advice to patients or accompanying person.	181 (92.8)
I instruct the patient to read a summary of the description of drug properties or other educational materials.	7 (3.6)
I do not advise about side effects and drug interactions.	7 (3.6)
**The most significant obstacle in advising about drug interactions in my daily practice is:**	
Lack of time.	84 (43.1)
Patient noncompliance	12 (6.2)
Lack of education in pharmacovigilance.	60 (30.8)
Lack of knowledge in rational pharmacotherapy.	19 (9.7)
None of the above because I do advise my patients about adverse effects and drug interactions in my daily practice.	20 (10.3)

Data are presented as numbers and percentages of participants based on questions regarding informing patients about drug interactions and side effects.

**Table 5:** Attitudes towards counseling about drug interactions and side effects.

	Number (%) of Correspondents				
	Fully Disagree	Disagree	Undecided	Agree	Fully Agree
Medical school gave me enough knowledge about drug side effects.	52	61	68	12	2
	(27)	(31)	(35)	(6)	(1)
Physicians specialists monitor and review the possibility of drug side effects when prescribing medications.	40	53	51	38	13
	(21)	(27)	(26)	(19)	(7)
I only tell patients about side effects if they ask me.	53	61	58	18	5
	(27)	(31)	(30)	(9)	(3)
I do not think I should tell patients about side effects because it scares them.	63	43	64	22	3
	(32)	(22)	(33)	(11)	(2)
Knowledge about side effects and interactions makes my work in practice easier.	0	1	16	68	110
		(1)	(8)	(35)	(56)
Qualitative knowledge of drug side effects could significantly prevent patient mortality.	1	5	48	50	91
	(0.5)	(2.6)	(24.6)	(25.6)	(46.7)
The prevention of polypragmasia (simultaneous use of multiple medicines) is the responsibility of a family medicine practitioner.	29	30	48	41	47
	(14.9)	(15.4)	(24.6)	(21)	(24.1)
Medical school gave me enough knowledge about drug interactions.	56	68	57	11	3
	(28.7)	(34.9)	(29.2)	(5.6)	(1.5)
Physicians specialists monitor and review the possibility of drug interactions when prescribing medications.	34	60	54	33	14
	(17.4)	(30.8)	(27.7)	(16.9)	(7.2)
I only tell patients about interactions if they ask me.	59	49	59	19	9
	(30.3)	(25.1)	(30.3)	(9.7)	(4.6)
I do not think I should tell patients about drug interactions because it scares them.	64	52	57	18	4
	(32.8)	(26.7)	(29.2)	(9.2)	(2.1)
The multiple uses of drugs can be harmful to the patient.	2	10	39	52	92
	(1)	(5.1)	(20)	(26.7)	(47.2)
After the physician recommends the medication, I go through the possible side effects of this drug.	1	7	47	79	61
	(0.5)	(3.6)	(24.1)	(40.5)	(31.3)
After the physician recommends the medication, I go through the possible interactions of this drug.	0	10	47	81	57
		(5.1)	(24.1)	(41.5)	(29.2)
In elderly people, I try to cope every symptom with medication.	38	71	61	21	4
	(19.5)	(36.4)	(31.3)	(10.8)	(2.1)
I refer an elderly patient who takes more than eight types of medication to a clinical pharmacologist.	93	50	33	17	2
	(47.7)	(25.6)	(16.9)	(8.7)	(1)
I revise the list of medications in elderly patients every six months.	18	37	66	54	20
	(9.2)	(19)	(33.8)	(27.7)	(10.3)
I read articles about prevention of side effects of drugs my patients use.	16	33	66	68	12
	(8.2)	(16.9)	(33.8)	(34.9)	(6.2)
When prescribing medicine, I advise patient about side effects.	1	15	76	77	26
	(0.5)	(7.7)	(39)	(39.5)	(13.3)
If side effects occur, I’m trying to find out if the patient is using over-the-counter medication.	5	8	29	75	78
	(2.6)	(4.1)	(14.9)	(38.5)	(40)
In elderly patients that have warfarin in their therapy, I look at the summary of described characteristics of warfarin before I include a new medication.	9	22	42	81	41
	(4.6)	(11.3)	(21.5)	(41.5)	(21)

Data are presented as numbers and percentages of participants in relation to answers along the Likert scale based on questions regarding attitudes and practice in advising patients about drug interactions and side effects.

## References

[1] Milos V., Westerlund T., Midlöv P., Strandberg E.L. (2014). Swedish general practitioners’ attitudes towards treatment guidelines—A qualitative study. BMC Fam. Pract..

[2] Sharifi H., Hasanloei M.A., Mahmoudi J. (2014). Polypharmacy-induced drug-drug interactions; threats to patient safety. Drug Res..

[3] Magin P., Goode S., Pond D. (2015). GPs, medications and older people: A qualitative study of general practitioners’ approaches to potentially inappropriate medications in older people. Australas. J. Ageing.

[4] World Health Organization Data and Statistics for the Republic of Croatia. http://www.euro.who.int/en/countries/croatia/data-and-statistics.

[5] Köberlein J., Gottschall M., Czarnecki K., Thomas A., Bergmann A., Voigt K. (2013). General practitioners’ views on polypharmacy and its consequences for patient health care. BMC Fam. Pract..

[6] Beijer H.J.M., de Blaey C.J. (2002). Hospitalisations caused by adverse drug reactions (ADR): A meta-analysis of observational studies. Pharm. World Sci..

[7] Ko Y., Malone D.C., Skrepnek G.H., Armstrong E.P., Murphy J.E., Abarca J., Rehfeld R.A., Reel S.J., Woosley R.L. (2008). Prescribers’ knowledge of and sources of information for potential drug-drug interactions: A postal survey of US prescribers. Drug Saf..

[8] Katic M., Juresa V., Oreskovic S. (2004). Family medicine in Croatia: Past, present, and forthcoming challenges. Croat. Med. J..

[9] Vojvodic Z., Nelken-Bestvina D., Kurc-Bionda A., Stimac D. (2014). Trends in prescribing in primary care in Croatia, 2000–2012: Prescribing volume, costs and regulatory measures. Coll. Antropol..

[10] Remesh A. (2012). Identifying the reasons for under reporting of ADR: A cross sectional survey. Res. J. Pharm. Biol. Chem. Sci..

[11] Gupta P., Udupa A. (2011). Adverse drug reaction reporting and pharmacovigilance: Knowledge, attitudes and perceptions among the resident doctors. J. Pharm. Sci. Res..

[12] Desai C.K., Iyer G., Panchal J., Shah S., Dikshit R.K. (2011). An evaluation of knowledge, attitude, and practice of adverse drug reaction reporting among prescribers at a tertiary care hospital. Perspect. Clin. Res..

[13] Alsaleh F.M., Alzaid S.W., Abahussain E.A., Bayoud T., Lemay J. (2017). Knowledge, attitude and practices of pharmacovigilance and adverse drug reaction reporting among pharmacists working in secondary and tertiary governmental hospitals in Kuwait. Saudi Pharm. J..

[14] HALMED Agencija Za Lijekove i Medicinske Proizvode. http://halmed.hr/Novosti-i-edukacije/Publikacije-i-izvjesca/Izvjesca-o-potrosnji-lijekova/Izvjesce-o-potrosnji-lijekova-u-Republici-Hrvatskoj-u-2016/.

[15] Schwarz E.B., Postlethwaite D.A., Hung Y.Y., Armstrong M.A. (2007). Documentation of contraception and pregnancy when prescribing potentially teratogenic medications for reproductive-age women. Ann. Intern. Med..

[16] Vranckx P., Valgimigli M., Heidbuchel H. (2018). The Significance of drug—Drug and drug—Food interactions of oral anticoagulation. Arrhythm Electrophysiol. Rev..

[17] Glassman P.A., Simon B., Belperio P., Lanto A. (2002). Improving recognition of drug interactions: Benefits and barriers to using automated drug alerts. Med. Care.

[18] Hema N.G., Bhuvana K.B. (2012). Pharmacovigilance: The extent of awareness among the final year students, interns and postgraduates in a government teaching hospital. J. Clin. Diagn. Res..

[19] Anthierens S., Tansens A., Petrovic M., Christiansens T. (2010). Qualitative insights into general practitioners views on polypharmacy. BMC Fam. Pract..

[20] Albrecht S. (2011). The pharmacist’s role in medication adherence. US Pharm..

[21] Yang S., Kim D., Choi H.J., Chang M.J. (2016). A comparison of patients’ and pharmacists’ satisfaction with medication counseling provided by community pharmacies: A cross-sectional survey. BMC Health Serv. Res..

[22] Karpa K.D., Hom L.L., Huffman P., Lehman E.B., Chinchilli V.M., Haidet P., Leong S.L. (2015). Medication safety curriculum: Enhancing skills and changing behaviors. BMC Med. Educ..

[23] Bokhof B., Junius-Walker U. (2016). Reducing polypharmacy from the perspectives of general practitioners and older patients: A synthesis of qualitative studies. Drug Aging.

[24] Gupta S.K., Nayak R.P., Shivaranjani R., Vidyarth S.K. (2015). A questionnaire study on the knowledge, attitude, and the practice of pharmacovigilance among the healthcare professionals in a teaching hospital in South India. Perspect. Clin. Res..

[25] Lavan A.H., Gallagher P.F., O’Mahony D. (2016). Methods to reduce prescribing errors in elderly patients with multimorbidity. Clin. Interv. Aging.

[26] Iyer S.V., Harpaz R., LePendu P., Bauer-Mehren A., Shah N.H. (2013). Mining clinical text for signals of adverse drug-drug interactions. J. Am. Med. Inform. Assoc..

[27] Rinner C., Grossmann W., Sauter S.K., Wolzt M., Gall W. (2015). Effects of shared electronic health record systems on drug-drug interaction and duplication warning detection. BioMed Res. Int..

[28] Dumic A., Miskulin I., Pavlovic N., Kenjeric D.C., Orkic Z., Miskulin M. (2018). Attitudes toward nutrition care among general practitioners in Croatia. J. Clin. Med..

